# Modulation of α_V_β_6_ integrin in osteoarthritis-related synovitis and the interaction with VTN_(381–397 a.a.)_ competing for TGF-β1 activation

**DOI:** 10.1038/s12276-021-00558-2

**Published:** 2021-02-01

**Authors:** Federica Ciregia, Céline Deroyer, Gaël Cobraiville, Zelda Plener, Olivier Malaise, Philippe Gillet, Marianne Fillet, Michel G. Malaise, Dominique de Seny

**Affiliations:** 1 Laboratory of Rheumatology, GIGA–I3, University of Liège, CHU de Liège, Liège, Belgium; 2 Department of Orthopedic Surgery, University Hospital Sart-Tilman, Liege, Belgium; 3 Laboratory for the Analysis of Medicines, CIRM, Department of Pharmacy, University of Liège, Liège, Belgium

**Keywords:** Osteoarthritis, Cell culture

## Abstract

Osteoarthritis is characterized by structural alteration of joints. Fibrosis of the synovial tissue is often detected and considered one of the main causes of joint stiffness and pain. In our earlier proteomic study, increased levels of vitronectin (VTN) fragment (amino acids 381–397) were observed in the serum of osteoarthritis patients. In this work, the affinity of this fragment for integrins and its putative role in TGF-β1 activation were investigated. A competition study determined the interaction of VTN_(381–397 a.a.)_ with α_V_β_6_ integrin. Subsequently, the presence of α_V_β_6_ integrin was substantiated on primary human fibroblast-like synoviocytes (FLSs) by western blot and flow cytometry. By immunohistochemistry, β_6_ was detected in synovial membranes, and its expression showed a correlation with tissue fibrosis. Moreover, β_6_ expression was increased under TGF-β1 stimulation; hence, a TGF-β bioassay was applied. We observed that α_V_β_6_ could mediate TGF-β1 bioavailability and that VTN_(381–397 a.a.)_ could prevent TGF-β1 activation by interacting with α_V_β_6_ in human FLSs and increased α-SMA. Finally, we analyzed serum samples from healthy controls and patients with osteoarthritis and other rheumatic diseases by nano-LC/Chip MS–MS, confirming the increased expression of VTN_(381–397 a.a.)_ in osteoarthritis as well as in lupus erythematosus and systemic sclerosis. These findings corroborate our previous observations concerning the overexpression of VTN_(381–397 a.a.)_ in osteoarthritis but also in other rheumatic diseases. This fragment interacts with α_V_β_6_ integrin, a receptor whose expression is increased in FLSs from the osteoarthritic synovial membrane and that can mediate the activation of the TGF-β1 precursor in human FLSs.

## Rheumatology: A starting point for joint disease

Insights into a mechanism underlying the formation of fibrotic tissue within joints in osteoarthritis may also prove relevant to other rheumatological disorders. The general mechanisms underlying fibrosis are reasonably well understood, but it remains unclear what triggers these processes in osteoarthitis. Researchers of the University of Liège in Belgium have uncovered a possible explanation based on experiments with cultured primary synovial fibroblasts from patients. Osteoarthitis is characterized by increased levels of a fragment of the protein vitronectin, and the researchers demonstrated that this in turn binds to a protein called α_V_β_6_, potentially promoting initiation of fibrosis. They also observed elevated levels of the same vitronectin fragment in two other rheumatoid disorders, lupus and systemic sclerosis, and concluded that further research is needed to characterize this protein’s role in inflammation and fibrosis.

## Introduction

Osteoarthritis (OA) is one of the most prevalent chronic joint diseases. This condition is more common in women and those over the age of 65, with a peak incidence around the age of 75 years^1,2^. Its prevalence is increasing worldwide due to the increasing rates of obesity and aging^1^. OA is characterized by structural alterations in the whole joint that typically exhibit cartilage degradation, subchondral bone sclerosis, and osteophyte formation. However, osteoarthritic synovium can also acquire an inflammatory phenotype, as mostly encountered in rheumatoid arthritis (RA), characterized by hyperplasia, leukocyte infiltration, neoangiogenesis, and fibrosis^3,4^. Cartilage and synovial fibrosis resulting from chronic inflammation and tissue injury have only been recently highlighted in OA^3,5,6^. Indeed, OA synovial tissue becomes rigid and thick, but the mechanisms that underlie fibrosis are still elusive. Synovial fibroblasts promote cell transformation into myofibroblasts expressing α-smooth muscle actin 2 (α-SMA) as well as excessive secretion of extracellular matrix (ECM) components^7^. TGF-β is one of the main players in fibrosis and is crucial for the fibrotic cascade activated in OA^6^. TGF-β is secreted in a latent form (pro-TGF-β), which is biologically inactive and associated with a noncovalent complex with the ECM. During biosynthesis, pro-TGF-β dimerizes through disulfide, which links to latent TGF-β-binding proteins (LTBPs) or glycoprotein-A repetitions predominant protein in large latent complexes. In response to injury, latent TGF-β complexes are modified into active TGF-β according to a tissue- and injury type-specific activation mechanism^8^. Integrin α_V_β_6_ is known to activate the latent form of TGF-β1. Integrins are receptors that connect the cytoskeleton to the ECM. They are heterodimers made up of noncovalently associated α and β subunits. In mammals, there are 18 different α subunits and 8 β subunits that can form 24 distinct heterodimers^9,10^. Integrins interact with different ligands (e.g., fibronectin and vitronectin (VTN)) that are mainly ECM molecules. They play an important role in cell adhesion but also in cell movement and migration. Integrins differ in tissue distribution and ligand identification. Recently, it has been reported that α_V_β_5_, α_V_β_3_, and α_V_β_6_ interact with PRGD_2_, and their expression levels are increased in osteoarthritic cartilage and osteophytes^11^. Integrin α_V_β_6_ interacts with molecules that contain the Arg-Gly-Asp (RGD) motif. Its formation is determined by β_6_ expression, since it can form a dimer only with α_V_, while α_V_ can interact with other β subunits (i.e., β_1_, β_3_, β_5_, and β_8_)^12^. Integrin α_V_β_6_ is known to be involved in the activation of TGF-β1 by interacting with the latency-associated peptide (LAP) of TGF-β1 through its RGD motif^13^. Integrin α_V_β_6_ plays a role in fibrosis, as suggested by Munger and colleagues, who observed that mice lacking integrin α_V_β_6_ were protected from pulmonary fibrosis^13^. Indeed, in normal adult skin, kidney, lung, and liver, epithelial cells have low or absent basal expression of α_V_β_6_ integrin, while it is upregulated in inflammation, cancer, wounds, and fibrosis^9,14^. In a previous study, we found increased levels of a V65 vitronectin fragment, VTN_(381–397 a.a.)_, in the synovial fluid and serum of OA patients with respect to controls (healthy and RA subjects)^15^. VTN is an ECM protein that participates in cell migration, adhesion, and spreading^16–18^. This protein interacts with α_V_-containing integrins and specifically with α_V_β_3_ integrin through its RGD motif^19^. VTN also plays a positive role in fibrosis^18,20,21^. Interestingly, the fragment VTN_(381–397 a.a.)_ is the result of VTN cleavage by plasmin, which affects the interaction of plasminogen activator inhibitor-1 (PAI-1) with VTN. Hence, PAI-1 is no longer trapped in the ECM, can inhibit plasminogen activation and may potentially mediate fibrosis^22,23^.

The aim of this work is therefore to investigate (i) whether VTN_(381–397 a.a.)_ can interact with integrin complexes, (ii) if α_V_β_6_ is expressed in synovitis in OA, (iii) if α_V_β_6_ can activate latent TGF-β1, and (iv) how VTN_(381–397 a.a.)_ can compete with TGF-β1. The presence of VTN_(381–397 a.a.)_ will also be investigated in other rheumatic diseases.

## Materials and methods

### Competition study

A competition study was performed to investigate the interaction of VTN_(381–397 a.a.)_ with five different integrins. Radiolabeled echistatin, a disintegrin known to bind integrin receptors, was used as a tracer for the binding assays. Lyophilized echistatin was provided by Bachem (Bubendorf, Switzerland) and radiolabeled by conjugation with a radioiodinated benzoate (^125^ISIB) to the epsilon amino group of lysine side chains. For the five integrins, α_5_β_1_, α_V_β_1_, α_V_β_3_, α_V_β_5_, and α_V_β_6_ (R&D System; Minneapolis, MN, USA), a solution at 1.8 µg/mL was prepared with integrin-binding buffer (IBB; 20 mM Tris pH 7.4, 150 mM NaCl, 2 mM CaCl_2_, 1 mM MgCl_2_, 1 mM MnCl_2_). Nunc Maxisorp Module plates were coated overnight at 4 °C with integrin complex at 1.8 µg/mL. After the wells were washed with sample buffer (IBB, 0.5% BSA), blocking solution (IBB, 2% BSA) was added, and the microplate was incubated for 2 h at room temperature. The wells were then washed three times with IBB/0.5% BSA. Then, ^125^ISIB-echistatin was diluted in sample buffer to fixed concentrations of 4, 4, 8, 8, and 20 nM for α_5_β_1_, α_V_β_1_, α_V_β_3_, α_V_β_5_, and α_V_β_6_ competition studies_,_ respectively. The ^125^ISIB-echistatin was mixed with sample buffer at a ratio of 1:1 (total binding = control well) or with increasing concentrations of ligands (Supplementary information SI1) [complete VTN, VTN_(381–397 a.a.)_, VTN_(365–381 a.a.)_] at a ratio 1:1 and added to wells in duplicate. We used 1,4,7-triazacyclononane-1,4,7-triacetic acid (NOTA) as a negative control. Then, 100 µL of prepared solutions (^125^ISIB-echistatin and ligands) was added to the plate and incubated for 2 h at room temperature with agitation. After rinsing, bound radioactivity was quantified using a gamma counter. The percentage of relative binding of radiolabeled echistatin was calculated as follows: radioactivity in the test well*100/radioactivity in the reference well. The reference well represents the total binding without ligand. For comparison of the potency of ligands in inhibiting bound ^125^ISIB-echistatin for different integrin complexes, the half-maximal inhibitory concentration (IC_50_) was calculated. The IC_50_ value was determined by constructing a dose–response curve and examining the effect of different concentrations of ligands on ^125^ISIB-echistatin binding. The experimental data were subjected to nonlinear regression using a five-parameter logistical model with GraphPrism 7 software.

### Primary cell culture and treatment

Synovial membranes were obtained from 22 OA patients (14 females, 8 males; mean age 69.8 ± 9.6 years, BMI 30.9 ± 6.8) during knee replacement, and primary synovial fibroblasts were isolated as explained previously^24^. The research ethics committee of CHU de Liege (Belgium) approved the study, and the patient gave informed consent to allow research procedures on samples. Human fibroblast-like synoviocytes (FLSs) were then cultured in DMEM supplemented with 10% FBS, 1% Pen/Strep, and 1% l-glutamine (all from Lonza; Basel, Switzerland) at 37 °C in an atmosphere of 5% CO_2_. In a plate, 5 × 10^4^ FLSs were stimulated with different compounds to analyze the modulation of α_V_β_6_: (i) TGF-β1, TNF-α, IL-1β, or IL-6 were used for 3 or 7 days at a concentration of 10 ng/mL; (ii) among the danger-associated molecular patterns, high-mobility group box 1 (HMGB1) was tested at 10, 50, and 100 ng/mL for 3 days, while S100A9 and S100A12 were tested at 100 and 200 ng/mL for 3 days or 24 h; (iii) advanced glycation end product (AGE-BSA) was tested at 50, 100, and 200 μg/mL for 3 days or 24 h; (iv) prednisolone and dexamethasone were used at 1 μM for 3 or 7 days; and (v) menadione or H_2_O_2_ was used to induce oxidative stress at 25–50–100 μM (3 h) and 100–200–400 μM (2 h), respectively, without FBS in the medium.

Finally, for analysis of the expression of α-SMA, FLSs (*n* = 5) were stimulated with VTN_(381–397 a.a.)_ (10, 25, 50, 100 ng/mL) with or without TGF-β1 (10 ng/mL) for 7 days.

### Western blot

The expression of α_V_ and β_6_ subunits and α-SMA in FLSs was evaluated by western blot analysis. Cells were scraped from plates and lysed with lysis buffer (25 mM HEPES, 150 mM NaCl, 0.5% Triton, 10% glycerol, 1 mM dithiothreitol) containing phosphatase inhibitors (25 mM β-glycerophosphate, 1 mM Na_3_VO_4_, and 1 mM NaF) and complete protease inhibitor mixture (Roche Applied Science; Penzberg, Germany). Total proteins were separated by 10% SDS–PAGE gels (12% for α-SMA) and transferred onto PVDF membranes. The membranes were then incubated with anti-α_V_ polyclonal antibody (1:250 dilution) (Cell Signaling Technology; Boston, MA, USA), anti-β_6_ polyclonal antibody (1:1000 dilution) (Abcam; Cambridge, UK) or anti-α-SMA monoclonal antibody (Agilent; Santa Clara, CA, USA; 1:1000 dilution). HRP-conjugated anti-rabbit antibody (1:1000 dilution) (Cell Signaling Technology) was used as the secondary antibody. Immunoblots were developed using the ECL chemiluminescent detection system. Rabbit anti-glyceraldehyde 3-phosphate dehydrogenase (GAPDH) (Merck) or rabbit anti-heat shock protein 90 (Hsp90; Santa Cruz) was used as loading controls at a dilution of 1:10,000 or 1:200, respectively. Values of optical intensity were normalized to GAPDH or Hsp90 levels. Data were analyzed by paired Wilcoxon test; a *p*-value < 0.05 was considered statistically significant. Statistical analysis was performed with GraphPad Prism 6 software (San Diego, CA, USA).

### Flow cytometry

Flow cytometry fluorescence-activated cell sorting (FACS) was applied to detect the presence of α_V_β_6_ integrin in FLSs. FLSs (*n* = 4) were detached by scraping and harvested on ice-cold PBS containing 1 mM MgCl_2_ and 0.1% BSA; 4 × 10^5^ cells were used for each condition. The cell scraper was used to prevent ablation induced by trypsin, and the experiment was performed on ice to prevent integrin internalization^25^. U87 glioblastoma cells that naturally expressed α_V_β_6_ integrin were used as a positive control^26^. Primary antibody recognizing α_V_β_6_ integrin (Merck; Darmstadt, Germany) was used at a concentration of 100 µg/mL in PBS/MgCl_2_/BSA. After incubation for 40 min on ice and two washes with PBS/MgCl_2_/BSA, the cells were incubated for 20 min on ice in the dark with Alexa Fluor 647-conjugated goat anti-mouse antibody (Thermo Fisher Scientific). After two washes, the cells were resuspended in 500 µL of PBS/MgCl_2_/BSA and filtered with a 100 µm strainer (Sysmex; Norderstedt, Germany) in FACS tubes; 1 µL of DAPI dye (Thermo Fisher Scientific) was added to identify dead cells. Flow cytometric measurements were performed with a FACSCanto^TM^ II (BD Biosciences; San Jose, CA, USA), and 10^4^ cells were analyzed in each assay. Flow cytometry standard files were then analyzed by FlowJo^TM^ software (BD Biosciences).

### Immunohistochemistry (IHC) of formalin-fixed paraffin-embedded tissues

Synovial biopsies (*n* = 23) were collected from synovial membranes provided from OA (*n* = 9), chronic pyrophosphate arthropathy (CPPA) (*n* = 7), and RA patients (*n* = 7) by needle arthroscopy from the affected knees (Table 1). These biopsies were fixed in 4% paraformaldehyde for 24 h at 4 °C, embedded in paraffin and characterized according to the histological inflammatory score (HIS) based on Tak’s score^27^. Briefly, HIS was obtained by hematoxylin-eosin staining measuring hyperplasia (0–4), infiltration of lymphocytes (0–4), plasma cells (0–4) and neutrophils (0–3) and by IHC for the infiltration of macrophages (CD68 staining, 0–3), leading to a total HIS of 18. The greater the score was, the greater the inflammatory status was.

**Table 1:** Clinical features of patients from whom synovial biopsies were collected after needle arthroscopy of the knee.

	OA	CPPA	RA
No.	9	7	7
Female (no.)	8	5	5
Age (median ± SD)	55 ± 14	65 ± 9	61 ± 17
BMI (median ± SD)	33 ± 7	24 ± 4	24 ± 5
ESR+	1/9	0/7	3/7
CRP+	2/9	3/7	6/7
RF+	0/9	0/7	2/7
Anti-CCP+	0/9	0/7	2/7
K&L score-median (interval)	3 (0–4)	2 (0–4)	n.d.
Histological inflammatory score median (interval)	4 (3–8)	5 (5–13)	14 (12–17)

*OA* Osteoarthritis, *CPPA* chronic pyrophosphate arthropathy, *RA* rheumatoid arthritis patients, *BMI* body mass index, *ESR* erythrocyte sedimentation rate, *CRP* C-reactive protein, *RF* rheumatoid factor, *anti-CCP* anti-cyclic citrullinated peptide, *K&L score* Kellgren and Lauwrence score peptide.

For IHC, slides were first incubated overnight at 65 °C. The day after, sections were dewaxed in xylene and subsequently passed through 100% ethanol and 70% ethanol. The unmasking was performed at 80 °C for 20 min. Endogenous peroxidase activity was then inactivated with 3% H_2_O_2_ for 20 min followed by blocking. Specific antibodies for β_6_ (Abcam) or α-SMA (Agilent) were used O/N diluted 1/500 or 1/50, respectively. Rinsed slides were incubated with HRP-labeled anti-rabbit antibody (Agilent) in a humidified chamber for 30 min at RT. Peroxidase was revealed with a Liquid DAB + Substrate Chromogen System (Agilent) for 10 min in a humidified chamber. Rinsed sections were counterstained for 30 s with Carazzy’s hematoxylin. Staining was detected with a Nanozoomer Digital Pathology 2.0 HT scanner (Hamamatsu Photonics, Hamamatsu, Japan). Images derived from IHC were analyzed with the bioimage analysis software QuPath^28^.

### TGF-β bioassay

Transformed mink lung epithelial cells (TMLCs), a generous gift from Prof. DB Rifkin (Department of Cell Biology, NYU School of Medicine, New York, NY), were used to test TGF-β1 functionality^29^. TMLCs were cultured in DMEM supplemented with 10% FBS, 1% Pen/Strep, and 1 mg/mL G418 Sulfate (Biowest; Riverside, MO, USA). For coculture, TMLCs were plated in 96-well plates at a density of 10^4^ cells per well, and fibroblasts were added at a density of 1.5 × 10^4^ cells per well, with a final volume of 100 μL. Cells were allowed to attach at 37 °C and 5% CO_2_. After 6 h, the medium was removed and replaced by the different treatments diluted in medium with 0.1% BSA without FBS (final volume of 100 μL).

For function-blocking experiments, cells were incubated with anti-α_V_β_6_ antibody (clone 10D5; Merck) or with the isotype control IgG2a kappa (clone eBM2a; Thermo Fisher Scientific) for 20 min at room temperature at a concentration of 50 μg/mL in medium containing 1 mM MgCl_2_/0.1% BSA before adding other compounds. Human latent TGF-β1 (hlatent TGF-β1; Cell Signaling Technology) was added at a concentration of 200 ng/mL^30^. For acidic activation, hlatent TGF-β1 was incubated for 10 min with 1 N HCl and neutralized by 1.2 N NaOH/0.5 M HEPES before it was added to cells. VTN_(381–397 a.a.)_ (Thermo Fischer Scientific) and hepcidin were used at 50 ng/mL. After 16 h of incubation at 37 °C and 5% CO_2_, the cells were washed with PBS and lysed by using 75 μL of cell culture lysis reagent (Promega, Madison, WI, USA) for 30 min at room temperature on a plate shaker. Luciferase activity was assessed by plate-reading luminometers with an injector (Victor™ X3; PerkinElmer Waltham, MA, USA) adding 100 µl of luciferase assay reagent (Promega) per well. Each condition was performed in triplicate for six different donors.

The data are expressed as relative luciferase activity (RLA). RLA is the measured luciferase activity of the coculture divided by the activity of the related control alone under the same conditions. Data were analyzed with the paired Wilcoxon test for non-normal data; a *p*-value < 0.05 was considered statistically significant. Statistical analysis was performed with GraphPad Prism 6 software.

### Serum collection

Thirty-eight healthy subjects and 38 OA patients were enrolled in the study to quantify the expression of the V65 fragment of VTN (_381_SQRGHSRGRNQNSRRPS_397_) or VTN_(381–397 a.a.)_. Blood samples were also collected from patients with different chronic inflammatory diseases: 46 with RA, 30 with ankylosing spondylitis (AS), 23 with systemic lupus erythematosus (SLE), and 20 with systemic sclerosis (SSc). OA patients were sorted into the early OA (*n* = 13) and late OA (*n* = 25) groups according to the K&L score. Data of the participants are summarized in Table 2. Human blood samples were collected under standard conditions and allowed to coagulate in plain glass tubes. Serum was obtained after centrifugation at 3000 rpm for 10 min at room temperature. Aliquots of supernatants were prepared and stored at −80 °C until use. The study was approved by the local institutional review boards of CHU Hospital of Liège.

**Table 2:** Clinical characteristics of patients enrolled in the study.

	HC	early OA	late OA	RA	AS	SLE	SSc
*n*	38	13	25	46	30	23	20
% Female	63	77	84	70	40	87	55
Age-median (range)	48 (24–72)	64 (51–77)	68 (61–86)	55 (17–77)	50 (23–71)	42 (17–68)	57 (37–83)
BMI-Median (range)	23 (19–28)	–	–	25 (16–40)	–	22 (17–31)	24 (19–32)
DAS (range)	–	–	–	4.2 (0.5–8)	–	–	–
ESR%	–	9	11	57	40	24	15
CRP+%	–	18	21	47	45	15	45
RF+%	–	0	0	63	–	–	–
Anti-CCP+%	–	0	0	85	–	–	–
HLA-B27+%	–	–	–	–	86	–	–

*HC* healthy control, *OA* osteoarthritis, *RA* rheumatoid arthritis, *AS* ankylosing spondylitis, *SLE* systemic lupus erythematosus, *SSc* systemic sclerosis, *BMI* body mass index, *DAS* disease activity score, *ESR* erythrocyte sedimentation rate, *CRP* C-reactive protein, *RF* rheumatoid factor, *anti-CCP* anti-cyclic citrullinated peptide, *HLA-B27* human leukocyte antigen.

### Nano-LC/Chip MS–MS

The levels of the V65 fragment of VTN in serum samples were analyzed as previously described^31^. Briefly, serum samples and calibration solution were purified and concentrated on an Oasis μElution WCX 96-well plate (Waters Corporation, Dublin, Ireland) before chromatographic separation and mass spectrometry analysis. ProtID-chip Zorbax 300SB (5 μm C18 phase, Agilent Technologies; Santa Clara, CA, USA) was used for chromatographic separation in gradient mode. A nanochip ESI source was operating in positive mode, and protonated peptide detection was performed by ion trap mass spectrometry (Agilent Technologies, Ion Trap LC/MS G6340A). The MS and MS/MS experimental parameters were optimized to be as sensitive and selective as possible. The intensities of the selected product ions were summed to extract ion chromatograms and subsequently integrated (QuantAnalysis software, Bruker Daltonik GmbH; Billerica, MA, USA). Area ratios (peptide *vs.* labeled peptide) were considered for quantitation. The linearity of the results for fragment quantitation was validated in the concentration range of 2.5–100 ng/mL, and the limit of detection was 0.76 ng/mL.

Differences in expression of VTN_(381–397 a.a.)_ among the examined classes of patients were analyzed with the unpaired Kolmogorov–Smirnov tests for nonparametric data.

## Results

### Integrin binding specificity of VTN_(381–397 a.a.)_

**Fig. 1:**
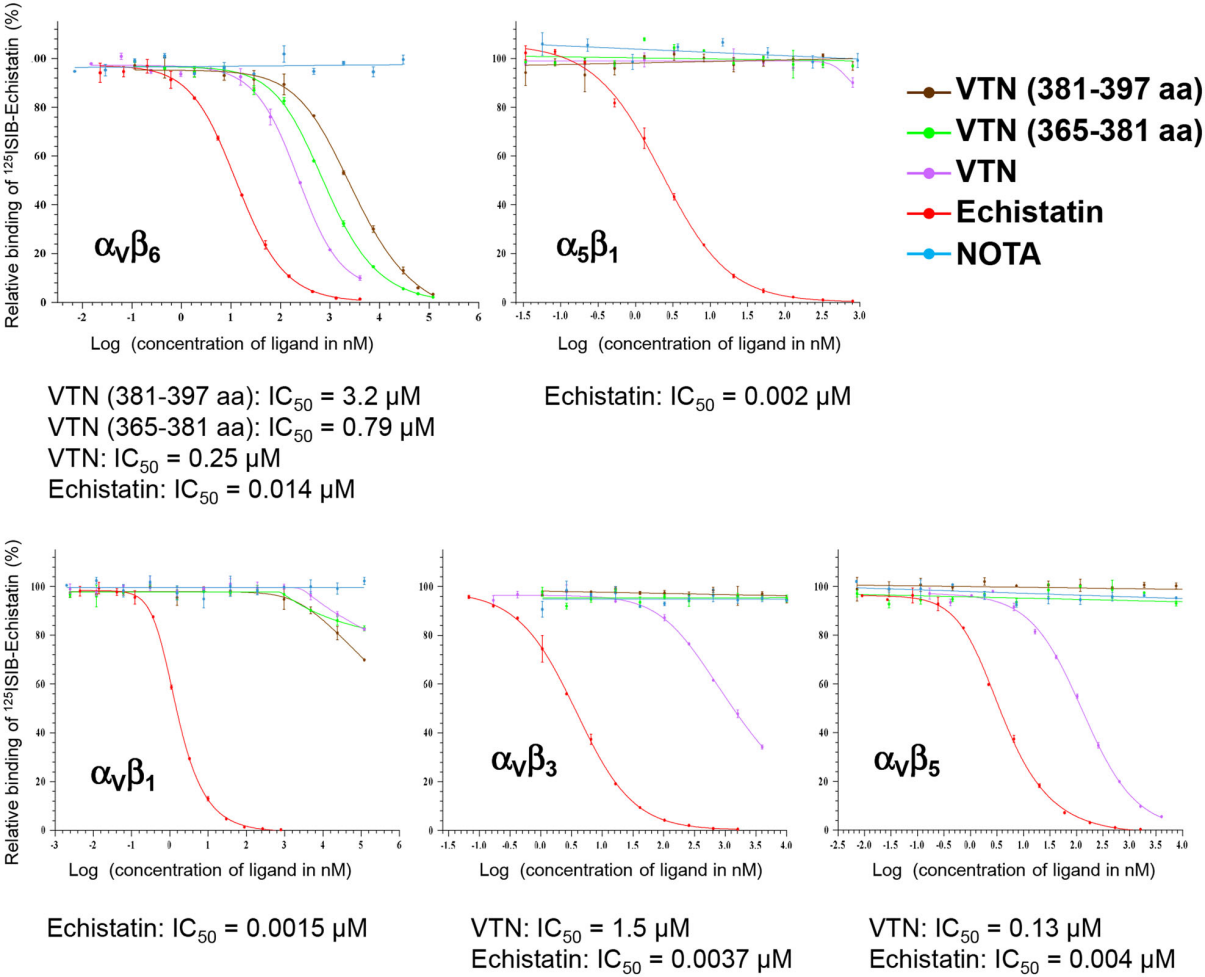
Results of the competition study. Competition curves between ^125^ISIB-echistatin and ligands on the recombinant human integrins α_V_β_6_, α_5_β_1_, α_V_β_1_, α_V_β_3_, and α_V_β_5_. The ligands were vitronectin (VTN) fragment 381–397 aa, VTN fragment 365–381 aa, and total VTN. NOTA was used as negative control. The IC_50_ values of ligands able to inhibit the echistatin interaction with recombinant human integrins are indicated.

To determine if VTN_(381–397 a.a.)_ could interact with integrins, we investigated its ability to compete with radiolabeled echistatin for binding to five different integrin complexes: α_5_β_1_, α_V_β_1_, α_V_β_3_, α_V_β_5_ and α_V_β_6_. We also tested the binding affinity of the whole VTN protein and another VTN fragment, VTN_(365–381 a.a),_ as previously described by Maile et al. ^32,33^. First, the binding affinity of echistatin to the five integrins was measured by determining the equilibrium dissociation constant (*K*_D_). *K*_D_ was evaluated by saturation assays involving addition of increasing concentrations of radiolabeled echistatin to a constant concentration of integrins (coated at 1.8 µg/mL). The *K*_D_ values calculated for each integrin complex were 1.48, 0.99, 3.17, 3.46, and 17.05 for α_5_β_1_, α_V_β_1_, α_V_β_3_, α_V_β_5_, and α_V_β_6_, respectively. Subsequently, competitive experiments were performed using increasing concentrations of ligand and a fixed amount of radiolabeled echistatin. The fixed concentration of radiolabeled echistatin was determined according to its *K*_D_ value. For each ligand concentration, the relative binding of ^125^ISIB-echistatin to coated integrins was then measured, and values were plotted versus the logarithm of ligand concentration. These competition curves together with the derived IC_50_ values are shown in Fig. 1. The VTN_(381–397 a.a.)_ peptide exhibited binding affinity only for integrin α_V_β_6_ (IC_50_ = 3.2 µM), as also observed with the other VTN_(365–381 a.a.)_ fragment (IC_50_ = 0.79 µM). The whole VTN protein also exhibited a high affinity for α_V_β_6_ (IC_50_ = 0.25 µM) but also for α_V_β_3_ (IC_50_ = 1.5 µM) and α_V_β_5_ (IC_50_ = 0.13 µM), as expected.

Therefore, considering that (i) the levels of cleaved VTN_(381–397 a.a.)_ fragments were increased in the serum and synovial fluid of OA patients^15^ and (ii) this fragment interacts with integrin α_V_β_6_, we investigated the expression of α_V_β_6_ integrin in OA synovial tissue.

### Integrin α_V_β_6_ expression in FLSs and OA-related synovitis

#### In vitro and in situ expression of α_V_ and β_6_ subunits or the α_V_β_6_ complex on FLSs

**Fig. 2:**
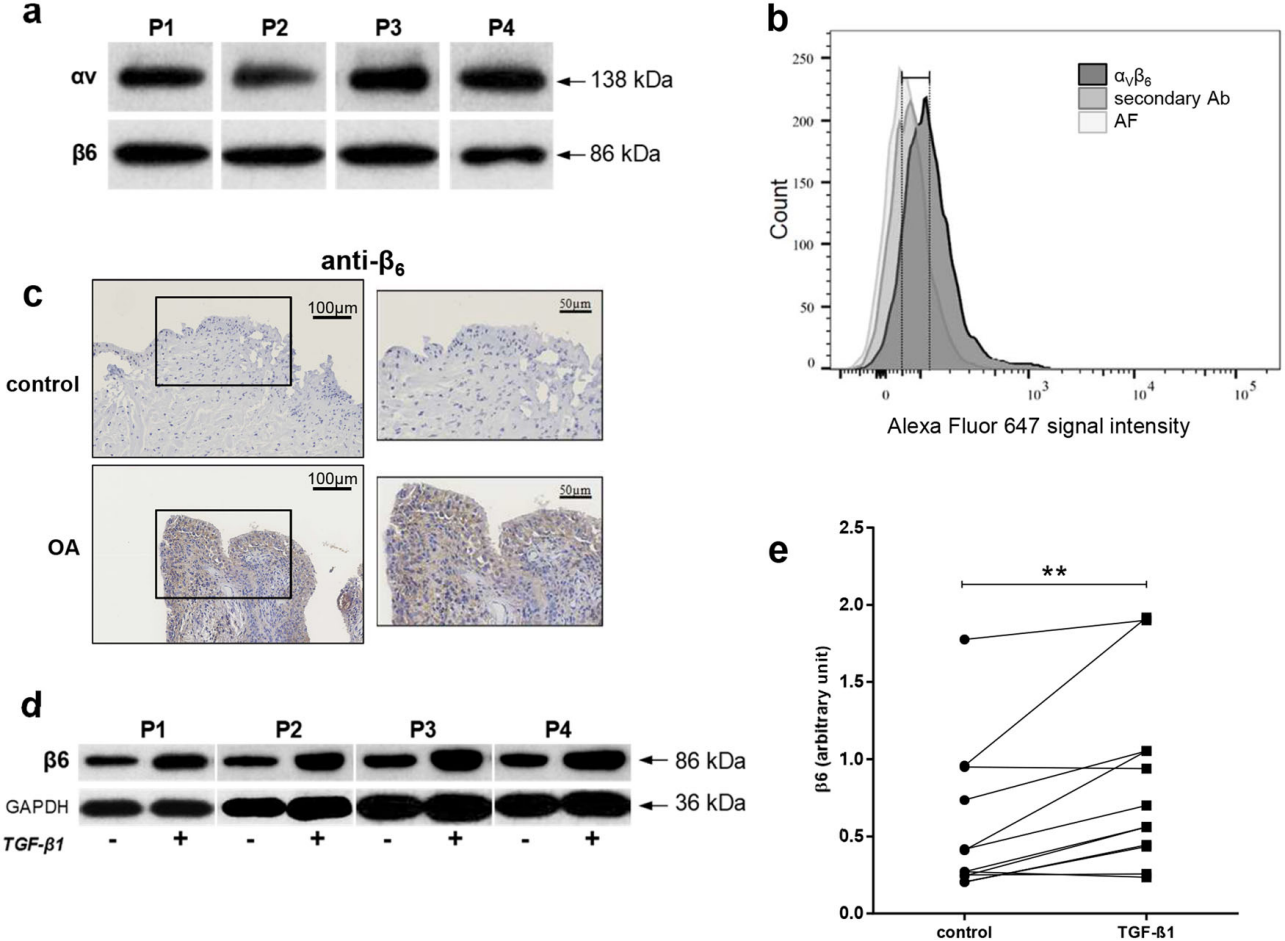
In vitro and in situ expression of the α_V_ and β_6_ subunits or the α_V_β_6_ complex on FLSs. **a** Expression of the α_V_ and β_6_ subunits shown by western blots of human fibroblast-like synoviocytes (FLSs) from four representative patients. **b** FACS histograms of flow cytometric analysis of FLSs from a patient with OA. Profiles of cell surface expression of the α_V_β_6_ integrin (dark gray) and controls: secondary antibody (Ab) alone (light gray) and autofluorescence (AF; lighter gray). This is the result from a representative patient; in supplementary information SI2, histograms from three other patients are shown. **c** Immunohistochemistry of human synovial membranes. Representative images of β_6_ in biopsies from a healthy donor (control) and an OA patient. **d** Representative images and quantification **e** of β_6_ signaling after TGF-β1 treatment. Values were normalized to that of glyceraldehyde 3-phosphate dehydrogenase (GAPDH). **p-value ≤ 0.01.

First, the expression of each subunit of the α_V_β_6_ integrin was observed by western blot analysis using human FLSs provided from the synovial membrane of OA patients (Fig. 2a). Second, the expression of the α_V_β_6_ integrin complex was determined by FACS analysis and illustrated by a flow cytometric histogram obtained with human FLSs previously incubated with α_V_β_6_ antibodies (Fig. 2b). As a negative control, FACS analysis was applied to FLSs incubated with only the secondary antibody (to exclude any nonspecific binding with the secondary antibody) and to FLSs with no antibody (to control the background derived from autofluorescence). Cells positive for DAPI were considered dying and were excluded from analysis by gating. The mean fluorescence intensity (MFI ± SD) obtained with human FLSs (*n* = 4) was significantly shifted from 53 ± 21 to 134 ± 29 in the presence of α_V_β_6_ antibody (*p*-value = 0.002). No significant variation in MFI was observed with the secondary antibody (Fig. 2b). However, it is noteworthy that strong basal autofluorescence of human FLSs was observed compared to control U87 cells (33 ± 3.2, data not shown). Supplementary information SI2 illustrates three other patients whose FLSs were analyzed by FACS.

These data confirmed the presence of the α_V_β_6_ integrin antibody on the cellular surface of the cellular membrane in FLSs from the OA patients. To confirm the presence of the α_V_β_6_ integrin in situ, we performed IHC analysis of synovial membranes provided from healthy individuals and OA patients and used an antibody directed against the β_6_ subunit (Fig. 2c).

#### Increased expression of the β6 subunit in FLSs and arthritic synovitis under profibrotic conditions

**Fig. 3:**
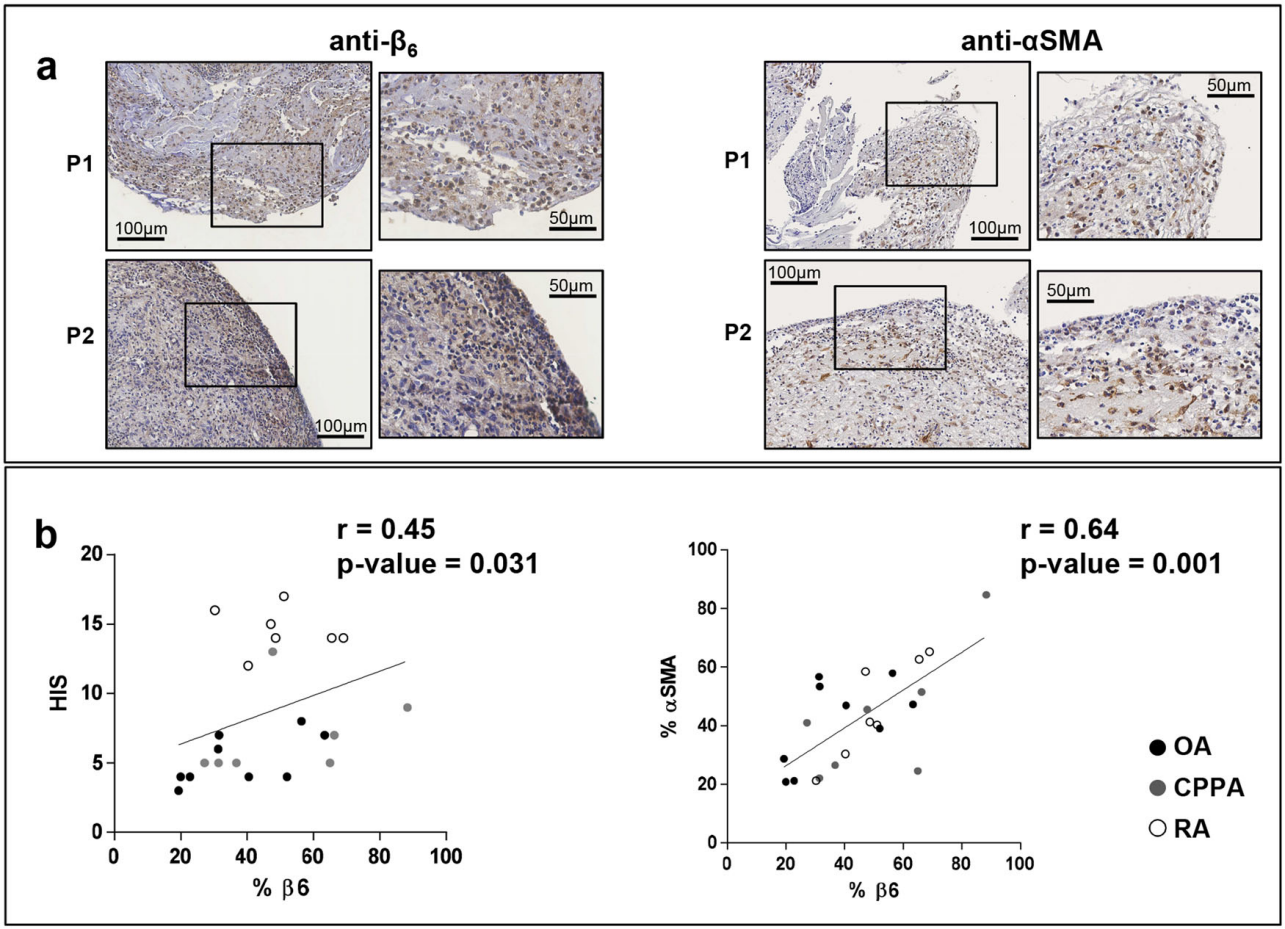
Expression of β_6_ in synovial membranes. **a** Immunohistochemistry on human synovial membranes. Representative images of β_6_ and α-SMA expression in biopsies from two different OA patients (P). **b** Spearman correlation analysis was applied to examine the correlation of the β_6_ percentage with the total histological inflammatory score (HIS) and α-SMA; *r* = Spearman coefficient. OA Osteoarthritis, CPPA chronic pyrophosphate arthropathy, RA rheumatoid arthritis.

We then wanted to evaluate whether the expression of the α_V_ and β_6_ subunits could be modulated in human FLSs by (i) a profibrotic mediator (TGF-β1), (ii) proinflammatory cytokines (TNF-α, IL-1β, and IL-6), (iii) danger-associated molecular patterns (HMGB1, S100A9, S100A12), (iv) advanced glycation end products (AGE-BSA), (v) drugs (prednisolone, dexamethasone), and (vi) oxidative stress (menadione, H_2_O_2_). We observed that none of these treatments influenced the expression levels of the α_V_ and β_6_ integrin subunits (Supplementary information SI3), except TGF-β1, which significantly increased β_6_ expression (*n* = 12) (Fig. 2d and e). Accordingly, β_6_ expression was evaluated in synovial tissues provided from the patients with OA (*n* = 9), CPPA (*n* = 7), and RA (*n* = 7). These biopsies were characterized by the α-SMA expression level, a fibrotic marker, as well as by HIS based on the following features: hyperplasia and infiltration of lymphocytes, plasma cells, neutrophils and macrophages, resulting in an HIS ranging from 0 to 18. The highest value of 18 represents the most inflamed synovitis. First, the β_6_ subunit was detected by IHC in 23 synovial biopsies. β_6_ staining was judged positive by the presence of brown staining in the cytoplasm (Fig. 3a). Then, with Spearman correlation analysis, a statistically significant correlation was observed between the β_6_ percentage and α-SMA expression (*r* = 0.64, *p*-value = 0.001) and, to a lesser extent, between the β_6_ percentage and HIS (*r* = 0.45, *p*-value = 0.031), confirming the in vitro results (Fig. 3b).

### Human FLSs activate human latent TGF-β

Integrin α_V_β_6_ is known to play a role in fibrosis^13^ and in the activation of latent TGF-β1, a profibrotic mediator once activated^34^. Based on our previous results, we wanted to confirm that (i) latent TGF-β1 could be activated in the presence of osteoarthritic FLSs and that (ii) the VTN_(381–397 a.a.)_ peptide could interact with α_V_β_6_.

**Fig. 4:**
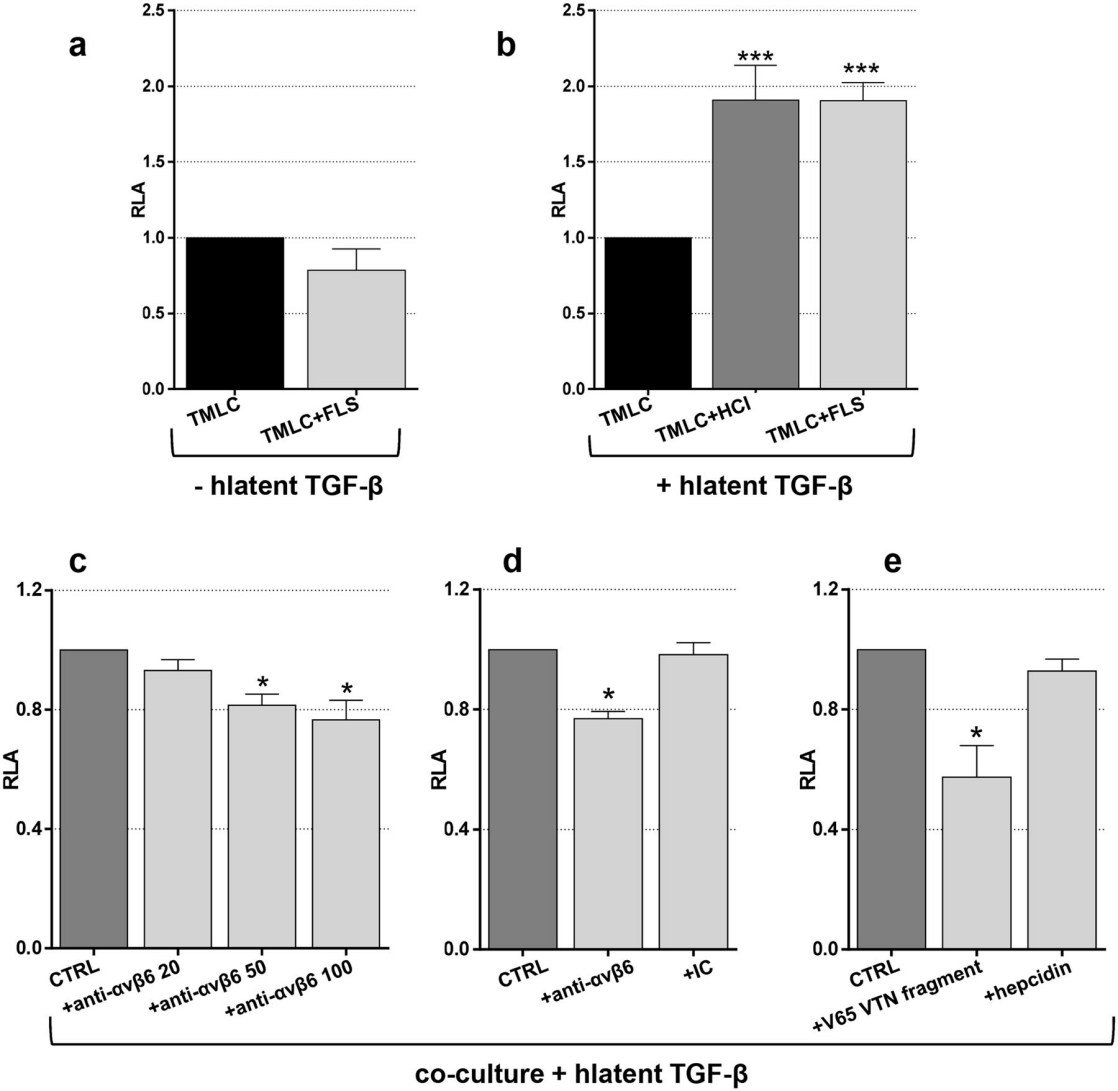
TGF-β luciferase bioassay. TMLCs and human FLSs were cultured for 16 h and lysed to measure luciferase activity. **a** Spontaneous production of active TGF-β: comparison between TMLCs and cocultures (TMLC + FLSs). **b** Activation of human latent TGF-β by acidic activation or by the presence of human FLSs. **c** and **d** Function blocking experiments with anti-α_V_β_6_ antibody. There was a reduction in TGF-β activation dependent on increasing concentrations of the anti-α_V_β_6_ antibody (20–50–100 μg/mL) (**c**). No reduction was observed with the isotype control (IC). IC and anti-α_V_β_6_ were used at 50 μg/mL (**d**). **e** Effect of the presence of the V65 vitronectin (VTN) fragment or peptide control (50 ng/mL). Human latent TGF-β was used at 200 ng/mL. The results are expressed as the relative luciferase activity (RLA) and are shown as the mean (±SEM). *p-value < 0.05; ***p-value ≤ 0.0001.

A quantitative TGF-β bioassay was applied to evaluate the activation of the TGF-β1 precursor based on its ability to induce PAI-1 expression. For this purpose, TMLCs were stably transfected with a construct containing the PAI-1 promoter fused to luciferase. In the presence of bioactive TGF-β1, increased PAI-1 expression resulted in a dose-dependent increase in luciferase activity in the cell lysates^29^. Low pH and interaction with integrin α_V_β_6_ are two mechanisms known to activate latent TGF-β1^35^. Therefore, to determine whether human FLSs can enhance TGF-β1 activation, we performed coculture with FLSs and TMLCs in the presence of latent TGF-β1. First, we observed that FLSs (*n* = 14) did not spontaneously produce a notable amount of active TGF-β1 in coculture, as shown in Fig. 4a. However, we observed a significant activation of latent TGF-β1 when FLSs were present, similar to TGF-β1 activation under acidic conditions (Fig. 4b). This finding strongly suggests that TGF-β1 activation is mediated by FLSs. Further, to confirm that α_V_β_6_ integrin was involved in this activation, we performed function-blocking experiments. Anti-α_V_β_6_ antibody was added to the coculture (*n* = 6), and a significant reduction in the activation of latent TGF-β1 was observed in a concentration-dependent manner (Fig. 4c). No significant variation was observed with the isotype control (Fig. 4d). Interestingly, when VTN_(381–397 a.a.)_ was added to the coculture (*n* = 6), the luciferase signal decreased, suggesting that this fragment can prevent the interaction of latent TGF-β1 with α_V_β_6_ integrin. No significant variation was observed with hepcidin, which was used as a control peptide (Fig. 4e). All the results are presented as the mean (±SEM) of RLA.

### Expression of α-SMA in FLSs

**Fig. 5:**
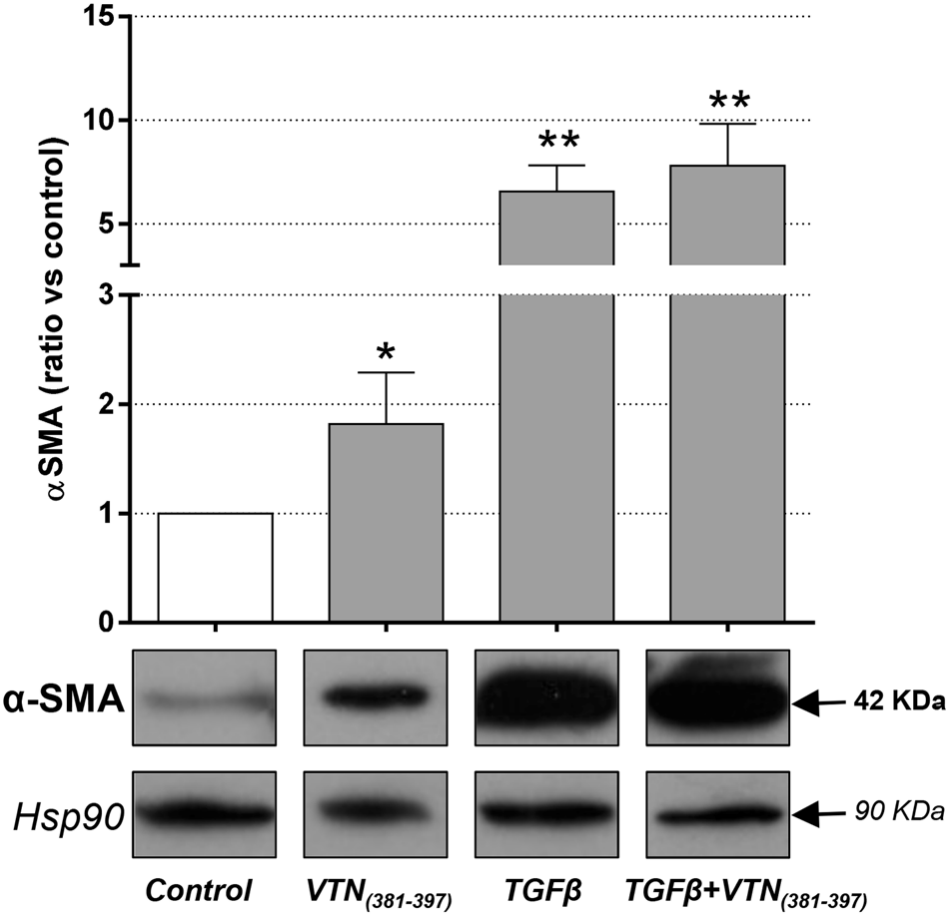
Expression of α-SMA in FLSs. Representative images and quantification of the α-SMA signal after treatment with VTN_(381–397)_ (10 ng/mL) with or without TGF-β1. Values were normalized to that of heat shock protein 90 (Hsp90). The results are expressed as the ratio of the normalized optical density *vs.* the control and are shown as the mean (±SEM). *p-value < 0.05; **p-value ≤ 0.01, *vs.* the control.

FLSs were stimulated with VTN_(381–397 a.a.)_ with or without TGF-β1 to evaluate the effect on the expression of the fibrotic marker α-SMA (Fig. 5). As expected, TGF-β1 upregulated the content of the fibrotic marker α-SMA and the presence of VTN_(381–397 a.a.)_ did not change this effect. Interestingly, when FLSs were treated with VTN_(381–397 a.a.)_ at 10 ng/mL, we observed a significant increase in α-SMA. Therefore, these preliminary results suggested a profibrotic effect for the VTN fragment. The results in Fig. 5 are expressed as the ratio of the normalized optical density *vs.* the control and are shown as the mean (±SEM).

### Quantification of VTN_(381–397 a.a.)_

**Fig. 6:**
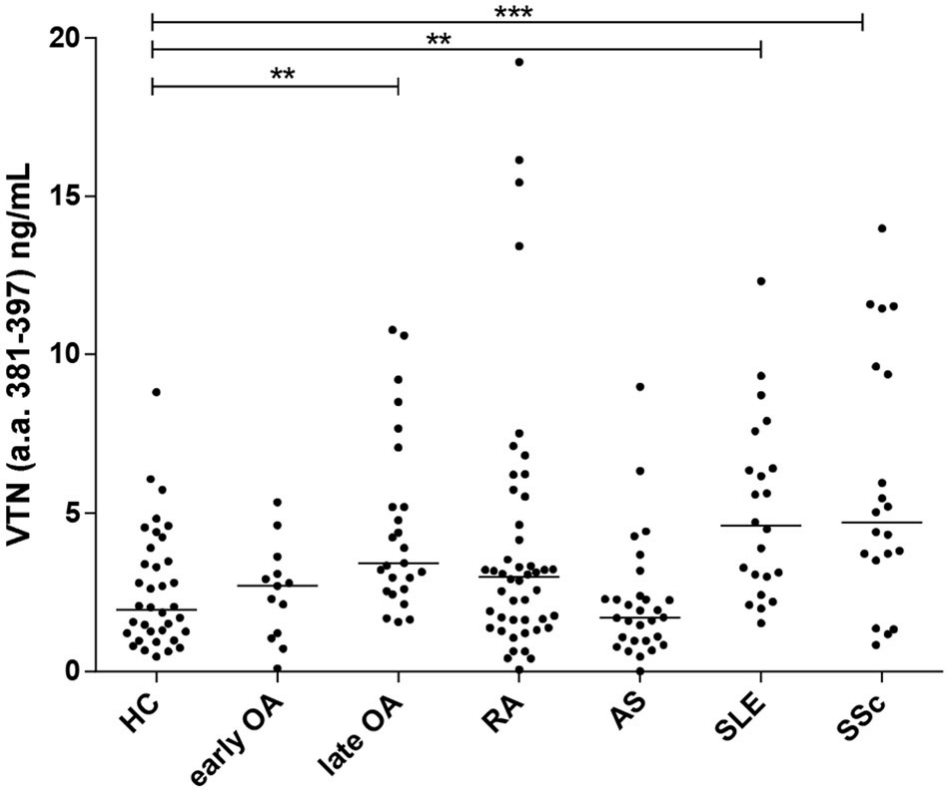
Nano-LC/Chip MS–MS. Quantification of VTN_(381–397 a.a.)_ by nano-LC/Chip MS–MS. Sera from patients with different chronic inflammatory diseases were analyzed. The median is indicated. *p-value ≤ 0.05; **p-value ≤ 0.01; ***p-value ≤ 0.001 (Kolmogorov–Smirnov test). Healthy control (HC), osteoarthritis (OA), rheumatoid arthritis (RA), ankylosing spondylitis (AS), systemic lupus erythematosus (SLE), systemic sclerosis (SSc).

To confirm the increased expression of VTN_(381–397 a.a.)_ in OA, we performed nano-LC/Chip MS–MS of serum samples from the healthy controls and the patients with OA or with different chronic inflammatory diseases: RA, AS, SLE, and SSc. We confirmed that the VTN_(381–397 a.a.)_ levels were increased in serum from the late OA patients compared to the healthy controls (*p*-value = 0.004). Furthermore, we observed a significant increase in this fragment in the patients with SLE and SSc compared to the healthy subjects, with *p*-values of 0.003 and <0.001, respectively (Fig. 6).

## Discussion

The present work relies on our previous proteomic studies, which highlighted the presence of a specific VTN fragment, VTN_(381–397 a.a.)_, in the serum and synovial fluid of patients with OA^15,31^. VTN_(381–397 a.a.)_ is a peptide composed of 17 amino acids at the C-terminal end of the V65 VTN subunit (a.a. 20–398) in the heparin-binding domain (HBD). VTN is a multifunctional ubiquitous glycoprotein produced by the liver that is secreted mostly as a serum protein and as a component of the ECM^36^. This molecule is also present in platelets and various human tissues^37^. This protein is involved in cell proliferation, differentiation, adhesion, and the immune response since it participates in fibrinolysis, coagulation, and the activation of the complement system^37–39^. Levels of VTN increased in pathological conditions related to acute inflammation such as RA and severe sepsis, where it seems to contribute to organ injury^39–41^. Moreover, its rising levels are considered a marker of fibrotic tissues (e.g., in liver and lung)^42–44^. Notably, VTN_(381–397 a.a.)_ is a cleaved fragment of VTN obtained by plasmin activation, reducing the affinity between VTN and PAI-1 and, consequently, fibrinolysis^22^.

In this study, we started to move towards a functional investigation of the role of VTN_(381–397 a.a.)_ in OA. VTN is known to interact with integrin receptors by its RGD motif, which is positioned far upstream from VTN_(381–397 a.a.)_ on the N-terminal end of VTN. However, Maile and colleagues also previously described a second integrin-binding site located within the HBD between amino acids 365 and 381^32,33^. Indeed, they demonstrated that VTN_(365–381 a.a.)_ could interact with α_V_β_3_ and that its binding was sufficient to enhance β_3_ phosphorylation^32^. Considering that the fragment VTN_(381–397 a.a.)_ belongs to the HBD, we hypothesized that it could interact with integrins through a different site from the RGD sequence. Therefore, through a binding competition assay, we explored the interaction affinity of VTN_(381–397 a.a.)_ with five different integrins: α_V_β_6_, α_V_β_3_, α_V_β_5_, α_V_β_1_, and α_5_β_1_. Whole VTN showed an affinity for α_V_β_5_ and α_V_β_6_ had a higher affinity for integrin α_V_β_3_, as previously described^9,19,45–48^. However, VTN_(365–381 a.a.)_ and VTN_(381–397 a.a.)_ only showed a high affinity for integrin α_V_β_6_ in our competition binding model.

Integrin α_V_β_6_ is known to be a key activator of TGF-β1 and plays a significant role in driving fibrosis (e.g., in liver, lung, and cancer)^9,49–51^. Indeed, the α_V_β_6_ integrin was markedly increased in pathological fibrosis and was suggested as a potential therapeutic target. TGF-β1 typically drives fibrotic processes^34^ but cannot be considered an antifibrotic target due to its ubiquitous role in tissue homeostasis. Fibrosis on synovial tissue was recently detected in OA and is considered one of the main causes of joint stiffness and pain^6^.

Thus, we have made progress by investigating the presence and regulation of this integrin in human FLSs from OA patients, which had never been observed before. Integrin α_V_β_6_ is a heterodimer of noncovalently associated α_V_ and β_6_ subunits. Using in vitro and in situ experiments, we observed the presence of the α_V_β_6_ integrin in osteoarthritic FLSs and synovial membranes. Although the presence of other integrins was previously described in FLSs^52^, this report is the first to show the presence of α_V_β_6_ in OA-related synovitis. By IHC, we also observed the expression of integrin β_6_ in biopsies of synovial membranes from the patients with OA, CPPA, and RA. β_6_ was positively correlated with the profibrotic marker α-SMA^7^ and, to a lesser extent, with the histological inflammatory score.

In addition, many different mediators of fibrosis, inflammation, and oxidative stress were tested in vitro to study the regulation of α_V_β_6_ expression. TGF-β1 was the only mediator able to increase β_6_ expression. Notably, the human FLSs used for this study were derived from OA patients, and the increase would have probably been more striking if we had assessed FLSs from healthy controls. Anyway, treatment with TGF-β1, a known regulator of fibrosis, induced a significant increase in the expression of the β_6_ subunit. TGF-β1 is required for the expression of *ITGB6* (β_6_ integrin gene) in epithelial cells, indicating mutual positive feedback between the two molecules^9,53,54^. TGF-β1 is produced as a pro-protein that dimerizes and links to LTBP. This inactive complex made of TGF-β1, LAP, and LTBP is referred to as a large latent complex^35^. Some authors found that organs have much more TGF-β1 precursor than could be required to trigger tissue fibrosis. This indicates that the function of this growth factor in fibrosis is mainly controlled by regulation of its bioactivation rather than its secretion or synthesis^55^. Therefore, we used the TMLC assay to detect the presence of bioactive TGF-β1 in cell coculture, and we observed that the presence of human FLSs could activate added latent TGF-β. The presence of an α_V_β_6_ blocking antibody in coculture significantly reduced the bioactivation of TGF-β1, supporting the hypothesis that α_V_β_6_ integrin is present on human osteoarthritic FLSs and that it can mediate TGF-β1 bioavailability. However, the presence of an α_V_β_6_ antibody in coculture did not completely abolish TGF-β1 bioactivation because other mechanisms are probably involved in the activation of latent TGF-β1^35^. Indeed, α_V_β_8_ is an integrin that has been increasingly studied in fibrosis for its role as a regulator of TGF-β1. Unlike α_V_β_6_ and other integrins, α_V_β_8_ appears to be only devoted to the activation of TGF-β1 and does not interact with the cytoskeleton^56,57^. We detected α_V_β_8_ expression in FLSs by western blotting (we used an anti-β_8_ monoclonal antibody, Abnova, Taipei, Taiwan; data not shown), and we also observed a significant reduction in the activation of latent TGF-β1 when an anti-β_8_ antibody was used in coculture of FLSs with TMLCs (monoclonal antibody, Abnova; Supplementary information SI4). Hence, the sole inhibition of the interaction of α_V_β_6_ with TGF-β1 cannot be sufficient to completely stop its bioactivation.

Moreover, considering that the premise of this study was the interaction of VTN_(381–397 a.a.)_ with the α_V_β_6_ integrin, we examined the effect of this fragment in vitro. Interestingly, when VTN_(381–397 a.a.)_ was added to cocultures, the luciferase signal significantly decreased, suggesting that this fragment could hamper the interaction of latent TGF-β1 with α_V_β_6_ integrin on human FLSs in OA. In this way, we also confirmed in vitro the VTN_(381–397 a.a.)_ affinity for α_V_β_6_ integrin as previously observed with the competition study.

Overall, this evidence could indicate a protective role of the fragment against fibrosis, considering the observed reduction in latent TGF-β1 activation. However, as described above, this reduction could be bypassed by other mechanisms, such as α_V_β_8_. We recently examined the mechanisms by which VTN_(381–397 a.a.)_ could be involved in OA. When FLSs were stimulated by VTN_(381–397 a.a.)_, the expression of the fibrotic marker α-SMA increased. Interestingly, this effect was observed at a concentration close to that found in plasma for pathological conditions. This result is consistent with previous findings proposing that VTN could exacerbate lung fibrosis through the upregulation of TGF-β1 signaling and the increase in α-SMA transcription^18^. Furthermore, it has already been proposed that TGF-β1 may induce myofibroblast differentiation by promoting differential interaction of integrins with ECM^58^ and that, in pulmonary fibrosis, fibroblasts express increased uPAR, which augments the binding of integrins to ECM proteins^59^. Thus, we could speculate that VTN_(381–397 a.a.)_ strengthens the profibrotic TGF-β1 pathway through α_V_β_6_. Future studies are clearly needed to more deeply define this role and interaction.

Finally, we aimed to substantiate previous findings^15^ by quantifying the presence of VTN_(381–397 a.a.)_ in serum from patients with OA and other inflammatory chronic diseases: RA, AS, SLE, and SSc. We applied a method of nanoliquid chromatography on chip tandem mass spectrometry that we formerly developed for the quantification of this specific fragment of VTN^31^. The increased expression of this peptide with respect to healthy subjects has been further confirmed in a new cohort of late OA patients but also in patients with SSc and SLE, while no rise was observed in patients with RA and AS. Hence, VTN_(381–397 a.a.)_ cannot be defined as a specific marker of OA, since it is present in other rheumatic diseases, but its presence can be related to the typical ECM alteration present in fibrosis.

In conclusion, these results corroborate our previous finding that VTN_(381–397 a.a.)_ expression levels are increased in the serum of OA patients but also in other rheumatic diseases, such as SSc and SLE. This fragment can interact with the α_V_β_6_ integrin, a receptor whose presence was confirmed on FLSs from OA patients and is involved in the activation of latent TGF-β1, possibly promoting fibrosis in OA. Taken together, these data warrant additional studies to unveil the fine molecular mechanisms that regulate the roles of VTN_(381–397 a.a.)_ and integrin α_V_β_6_. These findings will contribute to shedding light on the complexity of fibrosis in OA.

## Supplementary information

Supplementary Information

## Supplementary information

**Supplementary information** The online version contains supplementary material available at 10.1038/s12276-021-00558-2.
