# Supplementary material for: Modulation of αVβ6 integrin in osteoarthritis-related synovitis and the interaction with VTN(381–397 a.a.) competing for TGF-β1 activation
Source: Exp Mol Med. 2021 Feb 1;53(2):210–22. doi: 10.1038/s12276-021-00558-2 (PMC8080589; doi:10.1038/s12276-021-00558-2)
Supplement: Supplementary file 1 — Supplementary Information [file 12276_2021_558_MOESM1_ESM.pdf]

Supplementary information SI1.

Concentration range of 5 ligands diluted in sample buffer (final concentration on the plate) according to coated integrin complex.

Ligand	$\alpha_5\beta_1$	$\alpha_v\beta_1$	$\alpha_v\beta_3$	$\alpha_v\beta_5$	$\alpha_v\beta_6$
**$VTN_{(381\text{-}397a.a.)}$**	0.8 µM to 0.034 nM	120 µM to 2.46 pM	25 µM to 1.05 nM	30 µM to 7.15 pM	120 µM to 0.11 nM
**$VTN_{(365\text{-}381a.a.)}$**	0.8 µM to 0.034 nM	120 µM to 2.46 pM	25 µM to 1.05 nM	30 µM to 7.15 pM	120 µM to 0.11 nM
**Human Vitronectin**	0.8 µM to 0.034 nM	4 µM to 0.082 pM	4 µM to 0.17 nM	4 µM to 0.17 nM	4 µM to 1.0 pM
**NOTA**	120 µM to 0.057 nM	120 µM to 2.46 pM	25 µM to 1.05 nM	30 µM to 7.15 pM	30 µM to 7.15 pM
**Echistatin**	0.8 µM to 0.034 nM	0.8 µM to 4.52 pM	1.6 µM to 0.067 nM	1.6 µM to 9.0 pM	4 µM to 0.023 nM

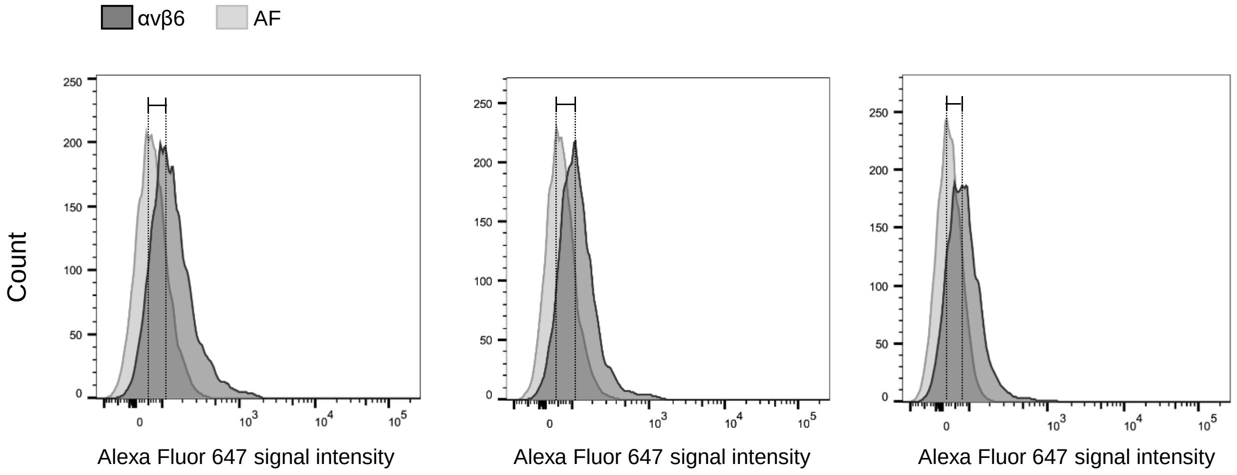

Supplementary information SI2.

FACS Histograms of flow cytometric analysis on FLS from 3 more patients with OA. Profiles of cell surface expression of $\alpha_V\beta_6$ integrin (dark grey) and auto-fluorescence (AF; lighter grey).

Supplementary information SI3.

Variation of α_V and β_6 expression in human fibroblast-like synoviocytes after stimulation with different compounds. Time of stimulation and employed substances are indicated. The graphs represent α_V and β_6 expression before and after stimulation, obtained by western blot. Values were normalized to GAPDH and analysed by paired Wilcoxon test.

α_V

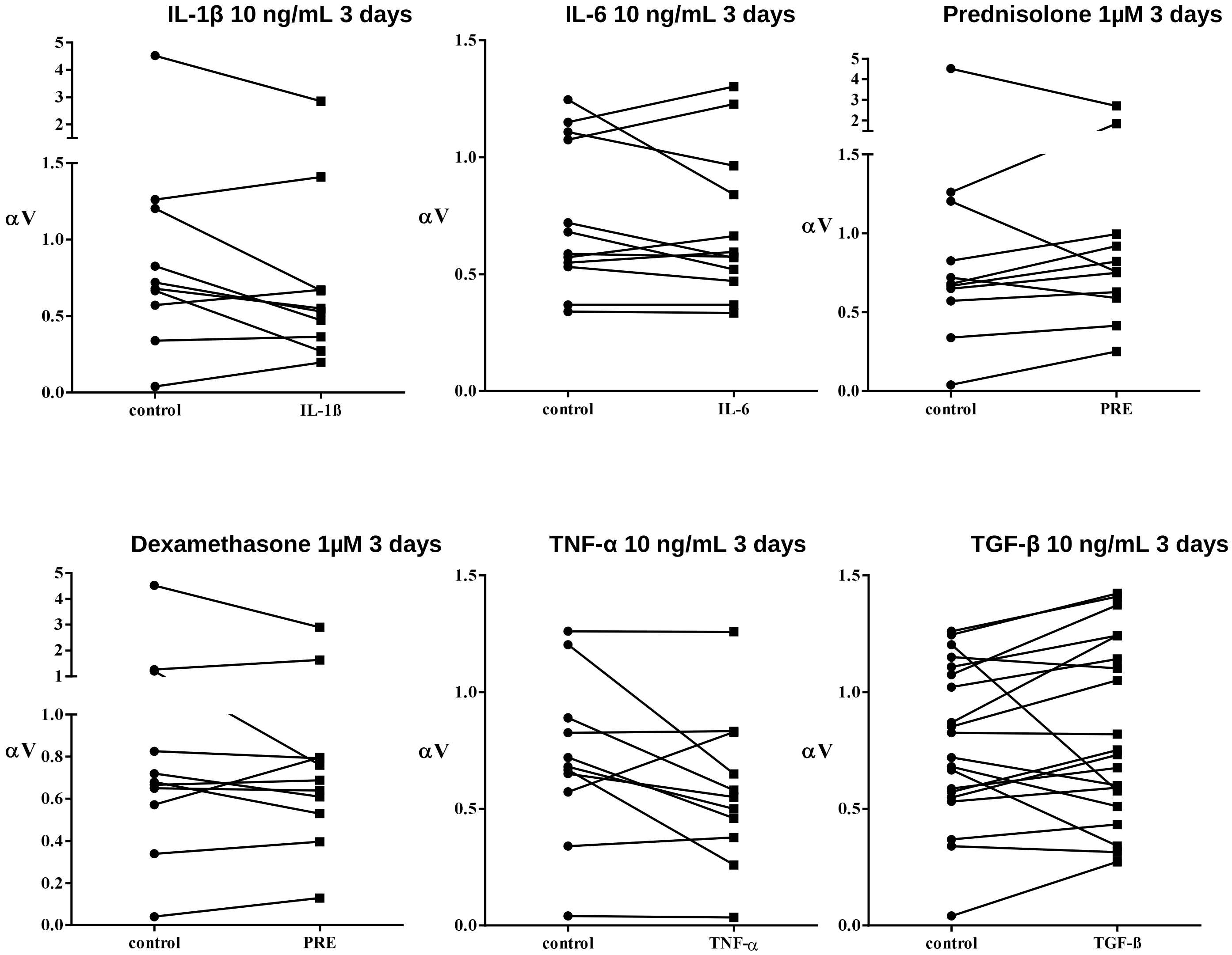

α_V

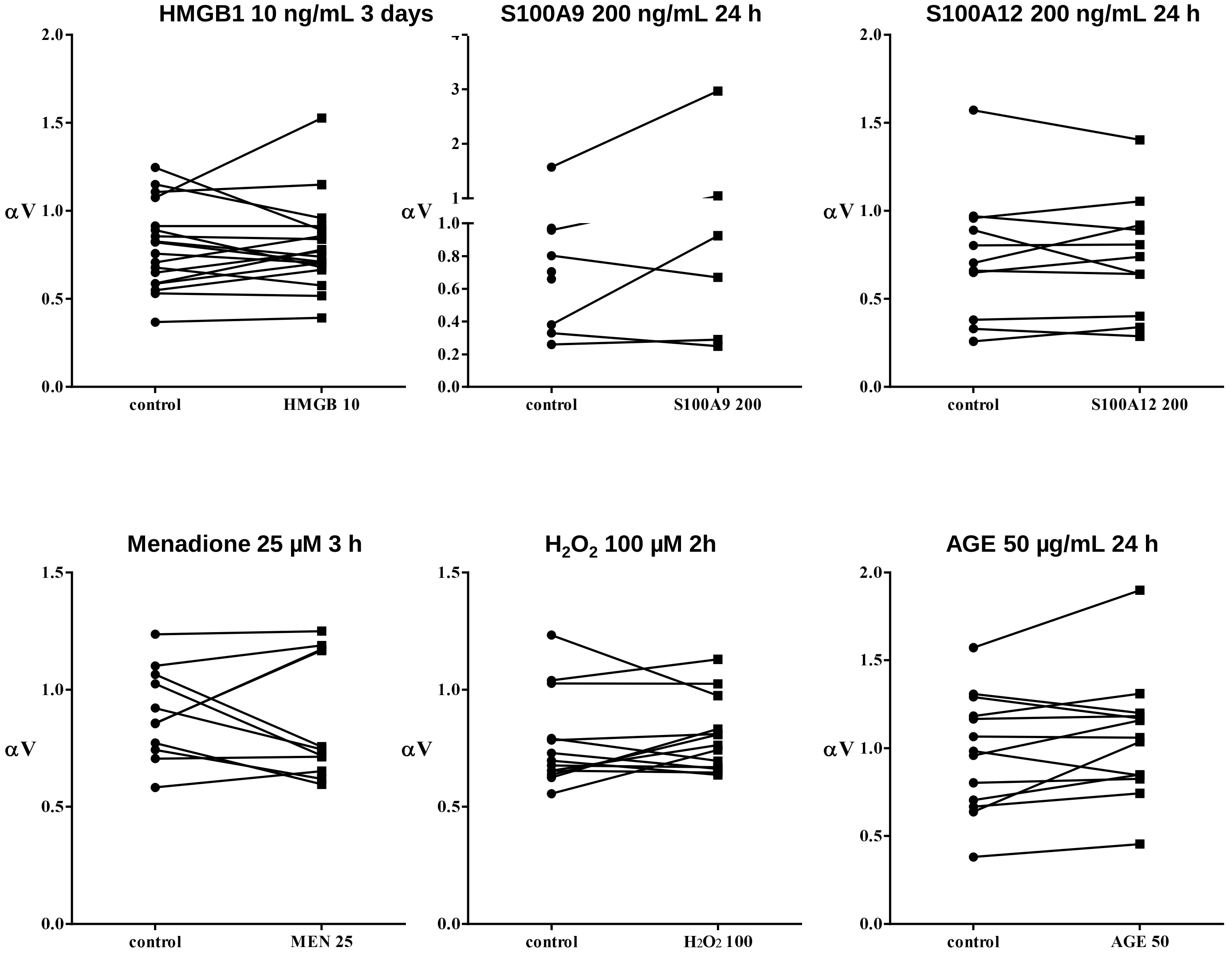

β_6

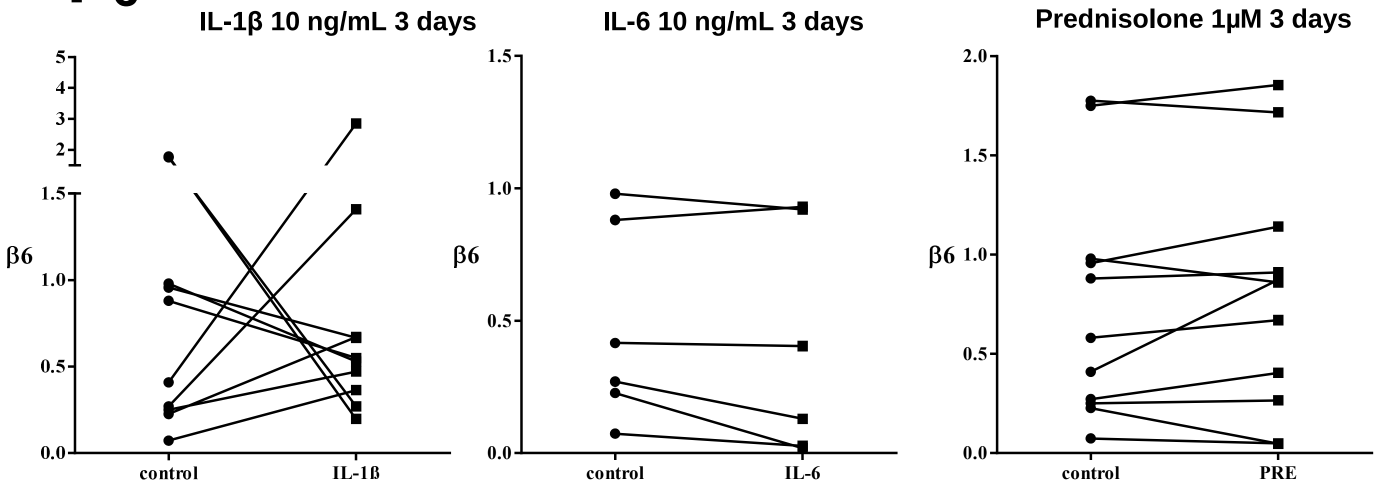

β_6

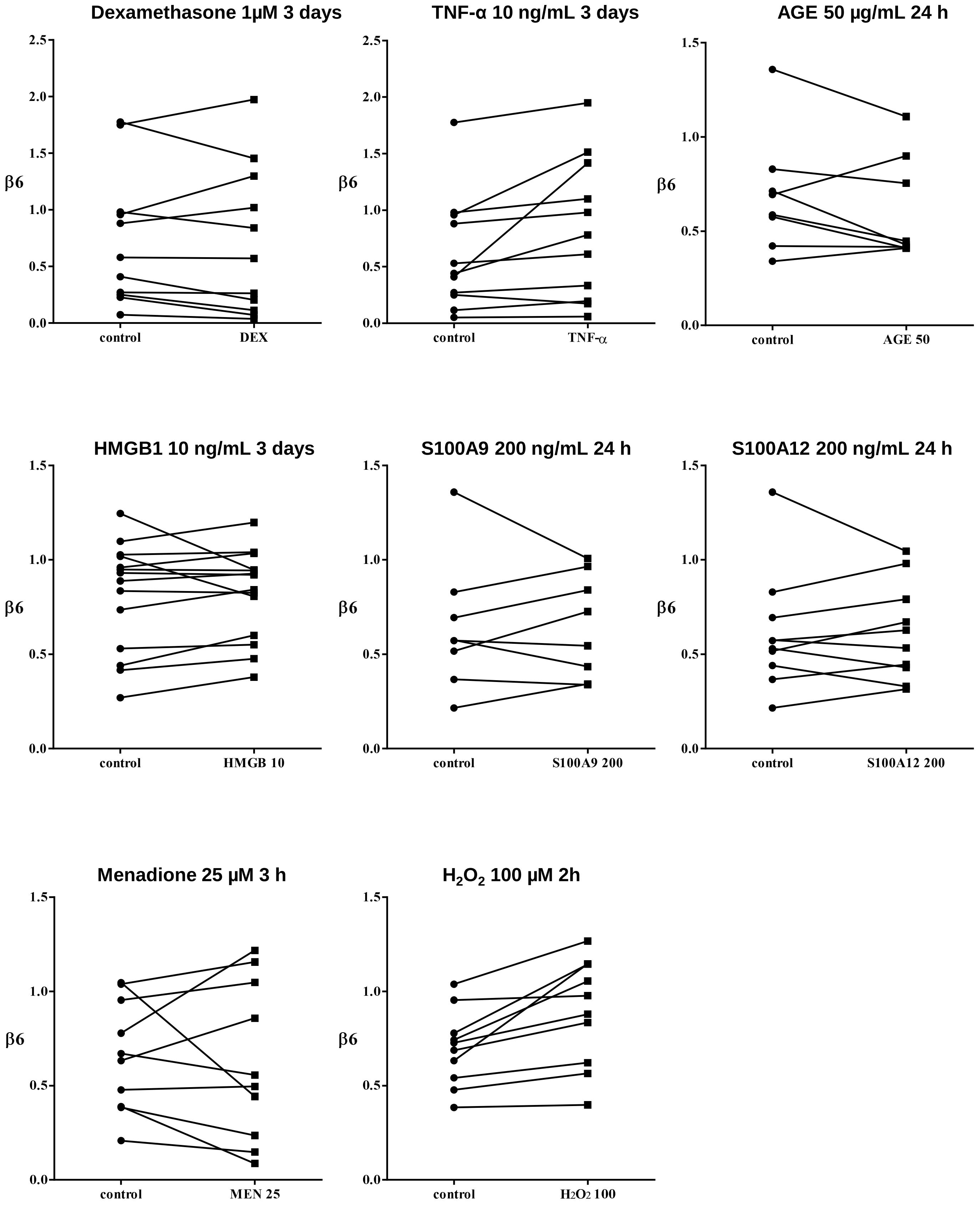
Dexamethasone 1µM 3 days
TNF-α 10 ng/mL 3 days
AGE 50 µg/mL 24 h
HMGB1 10 ng/mL 3 days
S100A9 200 ng/mL 24 h
S100A12 200 ng/mL 24 h
Menadione 25 µM 3 h
H2O2 100 µM 2h
β6
0.0
0.5
1.0
1.5
2.0
2.5
control
DEX
TNF-α
AGE 50
HMGB 10
S100A9 200
S100A12 200
MEN 25
H2O2 100

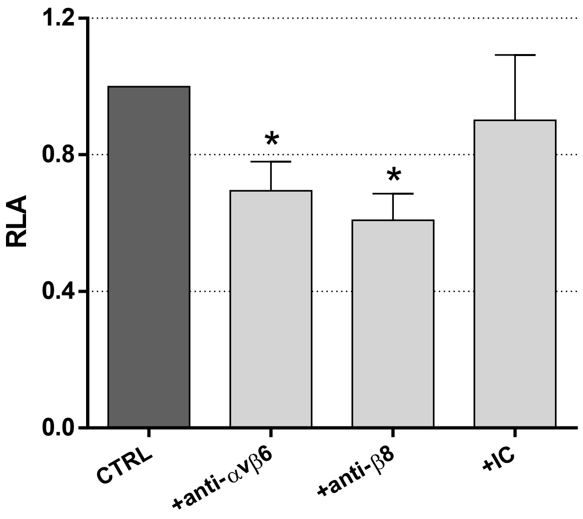

Supplementary information SI4.

Function-blocking experiments with $\alpha_V\beta_8$ integrin. To analyse if $\alpha_V\beta_8$ integrin was involved in TGF-β1 activation, function-blocking experiments were performed. Transformed mink lung epithelial cells (TMLC), were used for testing TGF-β1 activation. For co-culture, TMLC were plated in the 96-well plate at the density of 10^4 cells per well, and fibroblasts were added at the density of 1.5 x 10^4 cells per well. Cells were incubated with anti-$\alpha_V\beta_6$ antibody, anti-β_8 antibody or with the isotype control, for 20 minutes at room temperature at the concentration of 50 µg/mL before adding human latent TGF-β1 (200 ng/mL). Each condition was done in triplicate for 3 different donors. The data were expressed as relative luciferase activity (RLA) and analysed by Wilcoxon test. * p-value < 0.05 (*vs* CTRL).

A significant reduction in the activation of latent TGF-β1 was observed both with anti-$\alpha_V\beta_6$ antibody and anti-β_8 antibody, demonstrating that the bioactivation of TGF-β1 can occur by alternative pathways.
